# Predictors of severity and prolonged hospital stay of viral acute respiratory infections (ARI) among children under five years in Burkina Faso, 2016–2019

**DOI:** 10.1186/s12879-024-09219-x

**Published:** 2024-03-20

**Authors:** Abdoul Kader Ilboudo, Assana Cissé, Jennifer Milucky, Dieudonné Tialla, Sara A. Mirza, Alpha Oumar Diallo, Brice W. Bicaba, Kondombo Jean Charlemagne, Potiandi Serge Diagbouga, Daniel Owusu, Jessica L. Waller, Ndahwouh Talla-Nzussouo, Myrna D. Charles, Cynthia G. Whitney, Zekiba Tarnagda

**Affiliations:** 1 Laboratoire National de Référence-Grippes (LNR-G), Institut de Recherche en Sciences de la Santé (IRSS), Ouagadougou, Burkina Faso; 2 Direction de la Protection de la Santé de la Population, Ministère de la Santé, Ouagadougou, Burkina Faso; 3 National Center for Immunization and Respiratory Diseases, Centers for Disease Control and Prevention, Atlanta, GA USA; 4 Noguchi Memorial Institute for Medical Research, Legon, Accra Ghana; 5 Dexis Professional Services, 1331 Pennsylvania Avenue NW Suite 300, Washington, DC 20004 USA

**Keywords:** Acute respiratory infections, Severity, Prolonged length of stay, Predictors, Children under five, Burkina Faso

## Abstract

**Background:**

Viruses are the leading etiology of acute respiratory infections (ARI) in children. However, there is limited knowledge on drivers of severe acute respiratory infection (SARI) cases involving viruses. We aimed to identify factors associated with severity and prolonged hospitalization of viral SARI among children < 5 years in Burkina Faso.

**Methods:**

Data were collected from four SARI sentinel surveillance sites during October 2016 through April 2019. A SARI case was a child < 5 years with an acute respiratory infection with history of fever or measured fever ≥ 38 °C and cough with onset within the last ten days, requiring hospitalization. Very severe ARI cases required intensive care or had at least one danger sign. Oropharyngeal/nasopharyngeal specimens were collected and analyzed by multiplex real-time reverse-transcription polymerase chain reaction (rRT-PCR) using FTD-33 Kit. For this analysis, we included only SARI cases with rRT-PCR positive test results for at least one respiratory virus. We used simple and multilevel logistic regression models to assess factors associated with very severe viral ARI and viral SARI with prolonged hospitalization.

**Results:**

Overall, 1159 viral SARI cases were included in the analysis after excluding exclusively bacterial SARI cases (*n* = 273)very severe viral ARI cases were common among children living in urban areas (AdjOR = 1.3; 95% CI: 1.1–1.6), those < 3 months old (AdjOR = 1.5; 95% CI: 1.1–2.3), and those coinfected with *Klebsiella pneumoniae* (AdjOR = 1.9; 95% CI: 1.2–2.2). Malnutrition (AdjOR = 2.2; 95% CI: 1.1–4.2), hospitalization during the rainy season (AdjOR = 1.71; 95% CI: 1.2–2.5), and infection with human CoronavirusOC43 (AdjOR = 3; 95% CI: 1.2-8) were significantly associated with prolonged length of hospital stay (> 7 days).

**Conclusion:**

Younger age, malnutrition, codetection of *Klebsiella pneumoniae*, and illness during the rainy season were associated with very severe cases and prolonged hospitalization of SARI involving viruses in children under five years. These findings emphasize the need for preventive actions targeting these factors in young children.

**Supplementary Information:**

The online version contains supplementary material available at 10.1186/s12879-024-09219-x.

## Background

Despite the recent progress, acute respiratory infections (ARI) remain one of the leading causes of morbidity and mortality worldwide [1], with the highest burden found in Sub-Saharan Africa (SSA) and Southeast Asia [2]. ARI burden is particularly high in children under five, with an estimated 652,572 deaths occurring among this group in 2016 [2]. In children under five years of age, *Streptococcus pneumoniae* (also known as pneumococcus), respiratory syncytial virus (RSV), influenza virus, and *Haemophilus influenzae* type b (Hib) are some of the most common causes of ARI [3]. Specific interventions to improve the management of severe cases and effective vaccines against the leading etiologies have substantially reduced the burden of ARI in children [4]. The global use of routine vaccines against pertussis, selected pneumococcal serotypes, Hib, in many low and middle-income countries through their Expanded Programs on Immunization (EPI) has significantly reduced the burden of bacterial ARI in children under five. An estimated 17.7 million deaths among children less than five years of age have been prevented between 2011 and 2020 by effective vaccine administration in 73 countries supported by the GAVI Alliance [5].

Nevertheless, viral agents play an essential role in the morbidity and mortality caused by acute respiratory infections and might be appropriate targets for new vaccines. Several studies have identified viruses as causative pathogens of ARI [6–8]. In 2019, in a multicenter case-control study in children under five conducted in Africa and Asia, O’Brien et *al.* estimated viral etiologies to be responsible for about 60% of pneumonia hospitalization in children [9]. Other studies in Cameroon [10] and Burkina Faso [11] found a similar prevalence of viral pathogens in patients with ARIs. Despite the apparent predominance of viral etiology in ARIs, the study of factors associated with the severity of the disease is challenged by the difficulties of accurate routine etiological diagnosis, the multiplicity of involved pathogens, and the underlying biological complexity [9, 12]. In Sub-saharan Africa, the few studies that have explored the factors associated with ARI have identified malnutrition [13], the use of firewood as fuel for cooking and heating, and poor hygiene as increasing a child’s risk of ARI [14]. Details on the pathogens responsible for these infections and the simultaneous investigation for factors associated with the severity and mortality of viral ARI are found only in limited studies [15]. A series of studies conducted in Mali, Madagascar, and some American and Asian countries among children under five years of age found that *S. pneumoniae*, human metapneumovirus, respiratory syncytial virus, and influenza A viruses are the pathogens most associated with the severity and mortality of ARI [15–17]. These earlier studies indicate that viral ARIs are frequent and are major causes of morbidity and mortality, particularly among children under five years of age. However, the factors associated with severity and hospital length of stay are not well understood.

This study investigated the factors associated with the severity and longer hospital stay of ARI from common viral pathogens among children under five years of age by combining epidemiological and laboratory data (screening for respiratory microorganisms using rRT-PCR) from sentinel surveillance conducted at four health districts in Burkina Faso.

## Methods

### Study design and data collection

A cross-sectional study was conducted among children under five years of age admitted for severe ARI (SARI) in four public health facilities in Burkina Faso from October 2016 through April 2019. The study was implemented in the already existing framework of SARI sentinel surveillance in Burkina Faso, which included testing for influenza and other respiratory pathogens.

This surveillance was conducted inpatients of all ages receiving care at the National Teaching Hospital of Bogodogo, located in Ouagadougou, the capital city; Boussé District Hospital, located in the central region of the Plateau; Kongoussi District Hospital, located in the north-central region, and Houndé District Hospital located in the Hauts-Bassins region in the western part of the country. These sites were purposefully selected based on the following criteria: geographic representation, the high number of patients consulting at the health facility, the accessibility of the site to patients, the availability and the desire of the physicians or nurses to participate voluntarily in the surveillance program, and availability of a refrigerator (+ 4 °C) for storage of specimens.

Trained hospital health workers identified and enrolled patients meeting the 2014 World Health Organization (WHO) SARI case definition (an acute respiratory infection with a history of fever or measured fever ≥ 38 °C and cough with onset within the last ten days, requiring hospitalization) [18] and collected oropharyngeal (OP) and nasopharyngeal (NP) specimens at admission or during hospitalization [19]. In children under six months of age, only NP specimens were collected. Respiratory specimens were collected from all enrolled individuals, placed in a universal transport medium (Copan Diagnostics), stored at 4–8 °C, and transported to the national reference laboratory (Laboratoire National de Reference Grippes) within 48 h of collection for testing. The same staff collected socio-demographic and clinical data using a structured case report form.

### Study participants,case and variable definition

We included patients under five years of age who met the SARI case definition. We excluded children whose parents or legal guardians did not provide informed consent or whose medical conditions did not support specimen collection. For this report, we excluded children without (or with incomplete) laboratory results, those with exclusively bacterial or fungal pathogen detections, and those with unknown discharge status.

For this analysis, we defined a ***viral SARI (VSARI) case*** as an illness in any patient under five years of age meeting the inclusion criteria whose laboratory analysis of the OP/NP sample was positive for at least one of the following viral pathogens: influenza A, influenza B, influenza C; parainfluenza viruses 1, 2, 3, and 4; coronaviruses NL63, 229E, OC43, HKU1; human metapneumoviruses A and B; rhinovirus; respiratory syncytial viruses A and B; adenovirus; or enterovirus.

We defined a ***very severe viral ARI case (VSVARI)*** as any patient with viral ARI that required intensive care and/or any patient with at least one of the following danger signs: difficulty breathing, lethargy or coma, convulsion, stridor, inability to drink or eat, intercostal indrawing, and oxygen saturation under 90% [20].

Among the viral SARI cases, ***hospital stay*** was considered ***prolonged*** if the length of stay was equal to or greater than the mean length of stay of our study participants (seven days). Otherwise, it was considered short or normal.We used a modified version of the integrated age groups developed by the Eunice Kennedy Shriver National Institute of Child Health and Human Development (NICHD) in the United States [21] to categorize age into 0–3 month, 4–11 month, and 12–59 month. These age categorization aligns with the clinical practices observed in local hospitals and the particularity of acute respiratory infections in children.

### Laboratory testing and analysis

The national influenza reference laboratory received and tested the OP and/or NP specimens collected from enrolled cases using methods previously described by Cissé et *al.* [22]. Briefly, the specimens were screened individually to detect respiratory pathogens using eight multiplex real-time reverse-transcription polymerase chain reactions (rRT-PCR) with the FTD-33 Test Kit (Fast Track Diagnostics, Luxembourg). The pathogens identified included 21 types and subtypes of viruses, 11 types of bacteria, and one type of fungus: influenza A, influenza A subtype A(H1N1) pdm09, influenza B, and influenza C; parainfluenza viruses 1, 2, 3, and 4; coronaviruses NL63, 229E, OC43, and HKU1; human metapneumoviruses A and B; rhinovirus; respiratory syncytial viruses A and B; adenovirus; enterovirus; parechovirus; bocavirus; *Mycoplasma pneumoniae*; *Chlamydia pneumoniae*; *Streptococcus pneumoniae*; *Haemophilus influenzae*; *Haemophilus influenzae* type b; *Staphylococcus aureus*; *Moraxella catarrhalis*; *Bordetella* species (excluding *Bordetella parapertussis)*; *Klebsiella pneumoniae*; *Legionella* species; *Salmonella* species *and Pneumocystis jirovecii.*

### Statistical analysis

We used Epi-Info™ version 7.2.1.0 (CDC, Atlanta, GA, USA) for data recording and STATA® version 16.0 (StataCorp) for data cleaning and analysis. A series of bivariate analyses were performed between the dependent variables (very severe ARI, prolonged hospitalization) and the independent variables. The Chi-square test (χ^2^) or Fischer’s exact test was used to compare categorical variables, and the Student t-test or Mann-Whitney test was used to measure associations between qualitative and quantitative variables. A univariate logistic regression model was conducted to evaluate associations between dependent (outcome) and independent variables. Variables with a *p*-value ≤ 0.2 in the univariate analysis were entered into multivariable models through a backward stepwise elimination method to obtain the final model of variables. Key potential confounding variables (infection with RSV [12, 23], coinfection with *Streptococcus pneumoniae* and *Haemophilus influenzae b* [24]) were identified from the literature review and included in the model along with age even if the *p*-value was > 0.2. Simple and multilevel logistic regression models were used to assess factors associated with severity and prolonged hospitalization. The final models obtained were evaluated using different post-estimation tests available in STATA® version 16.0 (StataCorp) (Annex1 and 2 Figure S2, S3 and S4).

Because differences in care and discharge policies can affect the length of the hospital stay (common healthcare practices within the same hospital), we assumed that the length of hospital stay can be drove by clustering effect. We have then tested this grouped effect by running null two-effect with random intercepts and fixed effects model using as Level 1 the individual (children *n* = 905), and Level 2 corresponding the sentinel site (hospital *n* = 4). The test statistic was 139.45 with a corresponding *p*-value of less than 0.05 and so there was strong evidence that the between hospital variance is non-zero (Annex 1, Figure S1). Consequently, we conclude that accounting for group effects is justified. Crude and adjusted odds ratios (OR) along with 95% confidence intervals (95% CI) were estimated.

## Results

### Characteristics of the study population

**Fig. 1:**
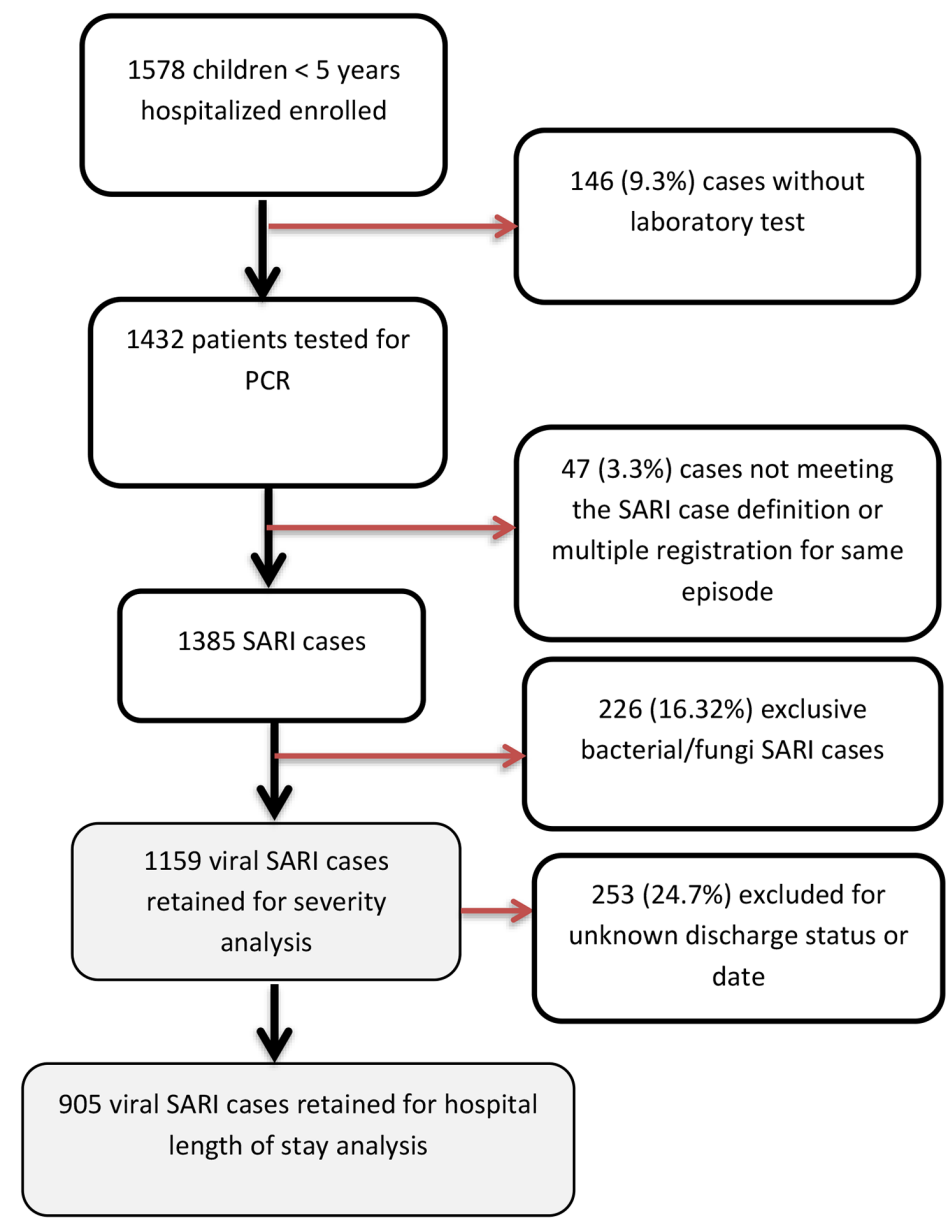
Cases selection diagram

The four sentinel surveillance sites identified and enrolled a total of 1578 children in the study. Of these, 146 (9.3%) did not provide specimens for laboratory testing and were excluded. Of the remaining 1432 patients who received laboratory testing, 47 (3.3%) did not meet the SARI case definition, and another 273 (19.1%) tested positive exclusively for bacterial or fungal infection and were excluded from the analysis. All cases were positive for at least one pathogen (virus, bacteria or fungus). Therefore, a total of 1159 (64%) viral SARI cases were included in severity analysis (Fig. 1). For the length of hospital stay analysis, 253 (24.7%) of viral SARI cases were excluded for unknown discharge status or date.

More than half of the participants were aged 12 to 59 months (56.3%), male (58.6%), and living in rural areas (55.7%). The majority (70.7%) were admitted in the dry season (November-April) [25]. A total of 344 participants (29.4%) met the definition of a very severe viral ARI (VSVARI) case (Table 1), while 214 (23.6%) had a prolonged hospital stay. The majority (85.8%) of the participants had viral and bacterial codetection (Table 2).

**Table 1:** Socio-demographic and clinical factors associated with severity of very severe viral acute respiratory infections (VSVARI) among children under five years of age

			Very severe viral acute respiratory infection compared to other SARI cases				
Variables		**Total sample ** *n* ** (%)** **Total ** *N* ** = 1159**	**Total** **VSVARI ** *n* ** (%VSVARI)** **Total ** *n* ** = 344**	**Crude OR** **(95% CI)**	***p-value***	**Adjusted** **OR (95% CI)**	***p-value***
Age group							
0–3 months		149 (12.9)	58 (16.9)	1.7 (1.2–2.4)	0.006**	1.5 (1.1–2.3)	0.044*
4–11 months		357 (30.8)	107 (31.1)	1.1 (0.8–1.5)	0.388	1.1 (0.8–1.5)	0.581
12–59 months		653 (56.3)	179 (52)	ref	---	1(ref.)	---
Area of residence							
Urban		514 (44.3)	166 (48.3)	1.25(1-1.6)	0.082	1.3 (1.1–1.7)	0.034*
Rural		645 (55.7)	178 (51.7)	1(ref.)	---	1(ref.)	---
Sex							
Male		679(58.6)	211(61.3)	1.17(0.9–1.5)	0.217	---	---
Female		480(41.4)	133(38.7)	1(ref.)	---	---	---
Season***							
Dry season		819(70.7)	220(70.7)	1(ref.)	--	1(ref.)	---
Rainy season		344(29.3)	124(340)	1.6(1,2–2)	0.001**	1.4(1.1–1.9)	0.028*
Sites							
Bogodogo		324(28)	73(21.2)	1(ref.)	--	---	---
Bousse		183(15.8)	49(14.2)	1.25(0.8–1.9)	0.283	---	---
Kongoussi		341(29.4)	60(17.4)	0.73(0.5–1.1)	0.112	---	---
Houndé		311(26.8)	162(47.1)	3.7(2.6–5.3)	< 0.001**	---	---
Antibiotics before admission							
No		460(39.7)	148(43)	1(ref.)	--	---	---
Yes		699(60.3)	196(57)	0.8(0.6–1.1)	0.132	---	---
Antibiotics during hospitalization							
No		19(1.6)	9(2.6)	2.2(0.9–5.4)	0.095	2.7(1.1–7.2)	0.048*
Yes		1140(98.4)	335(97.4)	1(ref.)	--	1(ref.)	---
Type of antibiotics used							
Ceftriaxone		1121 (96.7)	332(29.6)	0.9(0.4–1.8)	0.795	---	---
Penicilline G		12(1.04)	3(25)	0.8(0.2–2.9)	0.722	---	---
Gentamycin		104(9)	32(30.8)	1.1(0.7–1.6)	0.799	---	---
Others antibiotics****		29(2.5)	3(10.34)	0.3(0.1–0.9)	0.031*	---	---
Chronic health condition*****							
No		1147(99)	340(98.8)	1(ref.)	--	---	---
Yes		12(1)	4(1.2)	1.2(0.3-4)	0.776	---	---
Length of stay							
0–7 Days		920(79.4)	278(80.8)	1.1(0.8–1.5)	0.450	---	---
>7 Days		239(20.6)	66(19.2)	1(ref.)	--	---	---
Malnutrition							
No		1072(92.5)	323(93.9)	1.3(0.8–2.2)	0.247	1.6(0.9–2.8)	---
Yes		87(7.5)	21(6.1)	1(ref.)	--	1(ref.)	---

*p < 0.05 ** *p* < 0.01; VSVARI : Very severe viral acute respiratory infection ; ***Rainy season : June to September, dry season : October to may [25]; OR : odds ratio

****others antibiotics: Amoxicillin, metronidazole, ampicillin, cefotaxime; *****Chronic health conditions : asthma, circle cell diseases, HIV, diabetes, obesity, epilepsy, high blood pressure

**Table 2:** Prevalence of viral respiratory tract pathogens among viral SARI cases and association with very severe viral acute respiratory infections (VSVARI) among children under five years of age

		Very severe viral acute respiratory infection (VSVARI) compared with other viral SARI (VSARI) cases					
Variables		**Total positive (%)** **Total ** *N* ** = 1159**	**Total VSVARI** **(%VSVARI)** *N* = 344	**Crude OR** **(95% CI) (positive vs. negative)**	***p-value***	**Adjusted** **OR (95% CI)** (Positive vs. Negative)	***p-value***
Respiratory syncytial virus		212(18.3)	70(20.3)	1.2(0.9–1.7)	0.24	0.9(0.6–1.3)	0.526
Rhinovirus		449(38.7)	149(43.3)	1.3(1.01–1.7)	0.038*	---	---
Parechovirus		14(1.2)	5(1.5)	1.3(0.4-4)	0.62	---	---
Human parainfluenza virus 1		31(2.7)	3(0.9)	0.25(0.07–0.8)	0.022*	0.2(0.1–0.7)	0.009**
Human parainfluenza virus 2		21(1.8)	7(2)	1.2(0.5-3)	0.712	---	---
Human parainfluenzavirus 3		80(6.9)	30(8.7)	1.5(0.9–2.3)	0.114	---	---
Human parainfluenzavirus 4		57(4.9)	18(5.2)	1.1(0.6–1.9)	0.751	---	---
Influenza A		160(13.8)	35(10.2)	0.6(0.4–0.9)	0.021*	1.6(1.02–2.4)	0.04*
Influenza B		77(6.6)	16(4.7)	0.6(0.3–1.1)	0.080	---	---
Influenza C		15(1.3)	3(0.9)	0.6(0.16–2.1)	0.414	---	---
Human metapneumovirus		75(6.5)	28(8.1)	1.4(0.9–2.3)	0.135	---	---
Enterovirus		150(12.9)	49(14.2)	1.2(0.81–1.7)	0.391	---	---
Coronavirus OC43		43(3.7)	9(2.6)	0.6(0.3–1.3)	0.205	---	---
Coronavirus NL63		30(2.6)	13(3.8)	1.8(0.9–3.8)	0.102	---	---
Coronavirus HKU1		39(3.4)	6(1.7)	0.42(0.2–1.01)	0.054	---	---
Coronavirus 229E		11(0.9)	3(0.9)	0.9(0.2–3.3)	0.861	---	---
Bocavirus		118(10.2)	43(12.5)	1.4(0.9–2.1)	0.091	---	---
Adenovirus		256(22.1)	76(22.1)	1(0.7–1.3)	0.998	---	---
*Staphylococcus aureus*		205(17.7)	70(20.3)	1.3(0.9–1.8)	0.124	---	---
*Klebsiella pneumoniae*		385(33.2)	150(43.6)	1.9(1.4–2.5)	< 0.001**	1.6(1.2–2.2)	0.001
*Legionella pneumophil /Legionella longbeach*		1(0.1)	0(0)	---	---	---	---
*Streptococcus pneumoniae*		683(58.9)	224(65.1)	1.4(1.1–1.9)	0.006**	---	---
*Bordetella spp.*		5(0.4)	1(0.3)	0.6(0.06–5.3)	0.639	---	---
*Chlamydia pneumoniae*		2(0.2)	1(0.3)	2.3(0.14-38)	0.542	---	---
*Haemophilus influenzae*		516(44.5)	172(50)	1.36(1.1–1.8)	0.015*	---	---
*Haemophilus influenzae* type b		22(1.9)	13(3.8)	3.5(1.5-8)	0.004**	2.7(1.1–6.7)	0.03*
*Moraxella catarrhalis*		514(44.3)	173(50.3)	1.4(1.1–1.8)	0.008**	---	---
*Mycoplasma pneumoniae*		10(0.9)	3(0.9)	1.01(0.3–3.9)	0.982	---	---
*Pneumocystis jirovecii*		29(2.5)	11(3.2)	1.4(0.7–3.1)	0.327	---	---
*Salmonella spp*		2(0.2)	1(0.3)	2.4(0.15-38)	0.542	---	---
virus-bacteria codetection							
No		165(14.2)	28(8.1)	1(ref.)		---	---
Yes		994(85.8)	316(91.9)	2.3(1.5–3.5)	< 0.001**	---	---
Type of bacterial codetection							
No bacteria		165(14.2)	28(8.1)	1(ref.)		1(ref.)	
Mono-detection		251(21.7)	63(18.3)	1.6(1-2.7)	0.051	1.5(0.9–2.4)	0.152
Multiple bacterial codetection		743(64.1)	253(73.5)	2.5(1.6–3.9)	< 0.001**	2.2(1.4–3.5)	0.001**
Type of mixed viral infection							
Monoviral detection		584(50.4)	166(48.3)	1(ref.)		---	---
Two viruses		398(34.4)	122(35.5)	1.1(0.8–1.5)	0.451	---	---
Three and more viruses		176(15.2)	56(16.3)	1.2(0.8–1.7)	0.386	---	---

*p < 0.05 ** *p* < 0.01 VSARI : Viral severe acute respiratory infection VSVARI : Very severe viral acute respiratory infection OR : odds ratio

### Factors associated with very severe viral acute respiratory infections (VSVARI)

The adjusted odds of having very severe viral ARI (VSVARI) rather than VSARI were significantly greater in children under three months of age compared to those aged one year and more Adjusted odds ratio (AdjOR = 1.5; 95% CI: 1.1–2.3). In addition, urban residence has 1.6 greater odds of very severe viral SARI compared to residence in rural areas (AdjOR = 1.3; 95% CI: 1.1–1.7). Similarly, rainy season was associated with increased odds of very severe viral SARI compared to the dry season (AdjOR = 1.3; 95% CI: 1.1–1.7). Non-administration of antibiotic treatment during hospitalization was strongly associated with increased odds of very severe viral SARI cases in the multivariable analysis (AdjOR = 2.7; 95% CI: 1.1–7.2) (Table 1).

In univariate analysis, codetection of bacterial respiratory tract pathogens was associated with VSVSARI, specifically *S. pneumoniae*, *H. influenzae*, Hib, *Staphylococcus aureus*, *Moraxella catarrhalis*, and *Klebsiella pneumoniae*. The multivariable analysis confirmed the unadjusted effect of Human Para Influenza Virus 1 (unadjusted OR = 0.25 95% CI: 0.07–0.8) and Hib (unadjusted OR = 3.5(1.5–3.5) infection on the severity of viral ARI (Adj OR = 0.2; 95% CI:0.1–0.7), (Adj OR = 2.7(1.1–6.7) respectively and the risk associated with *Klebsiella pneumoniae* codetection (Adj OR: 1.6; 95% CI: 1.2–2.2) (Table 2).

### Factors associated with prolonged hospitalization for viral severe acute respiratory infections (VSARI)

**Fig. 2:**
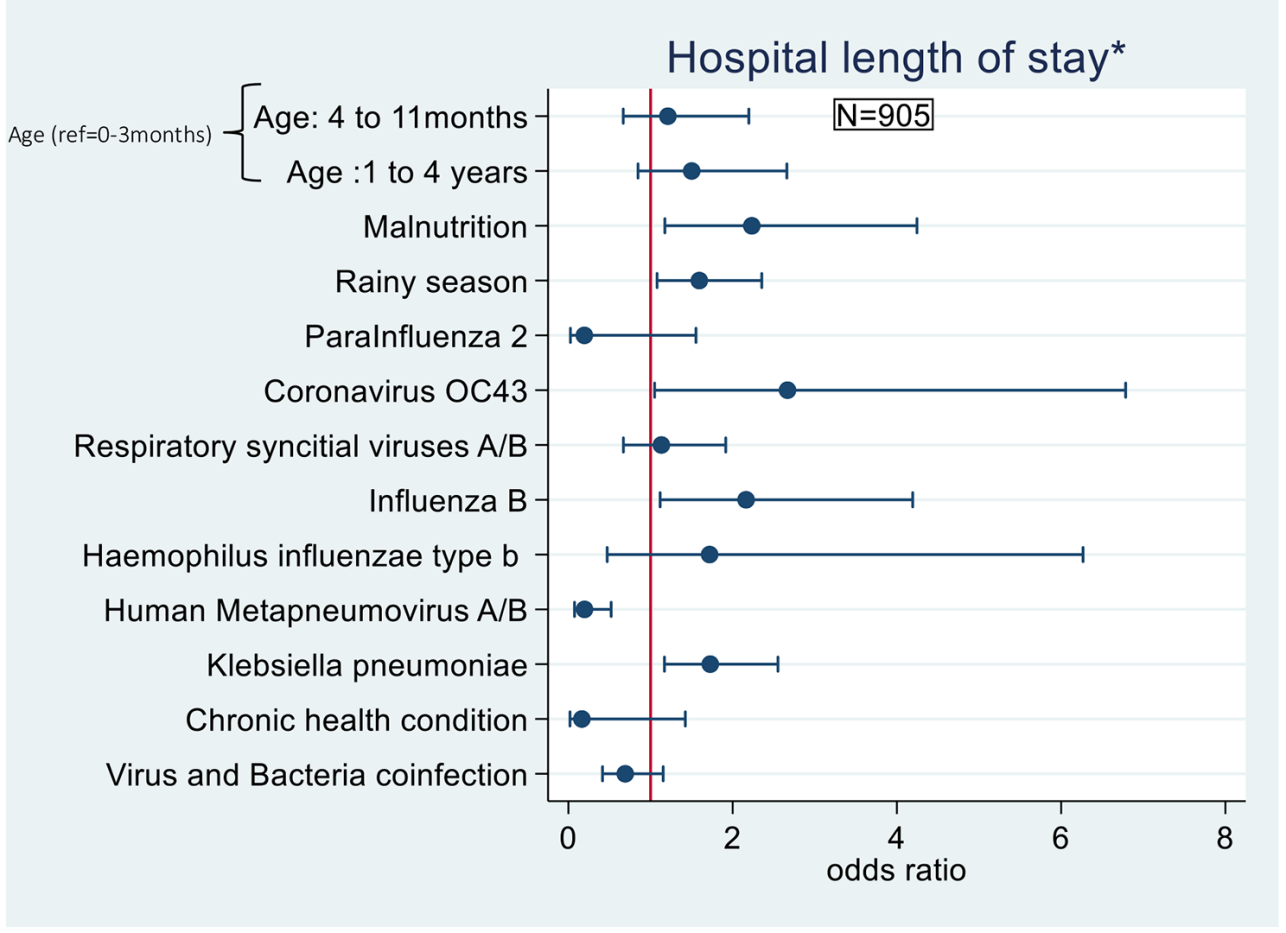
Factors associated with prolonged hospital stay of viral severe acute respiratory infections (VSARI) among children under five (multivariable analysis). *prolonged hospital stay = length of stay > 7 days;

In multivariable and multilevel analysis, malnutrition (estimated by using the definitions from UNICEF-WHO-World Bank [26]) was associated with increased odds of prolonged hospitalization compared to non-malnourished patients (AdjOR = 2.2; 95% CI: 1.2–4.2). Similarly, the odds of prolonged hospitalization for viral SARI were 1.6 times greater during the rainy season compared to the dry season (AdjOR = 1.6; 95% CI: 1.1–2.3) (Table 3). Furthermore, infection with human metapneumovirus was associated with decreased odds of prolonged hospitalization (Adj OR = 0.2;95% CI: 0.1–0.7). *Klebsiella pneumoniae* (AdjOR = 1.7; 95% CI: 1.2–2.5), influenza B (AdjOR = 2.2; 95% CI: 1.1–4.2), and coronavirus OC43 (AdjOR = 2.7; 95% CI: 1.1–6.8) were associated with increased odds of prolonged hospitalization (Table 4 and Fig. 2).

**Table 3:** Univariate analysis of socio-demographic and clinical factors associated with prolonged hospital stay (> 7 days) among viral severe acute respiratory infections (VSARI) among children under five years of age

Variables		Total (%) *N* = 905	Prolonged hospital**** stay (%) *N* = 214	Crude OR (95% CI)	*p*-value
Age group					
0–3 months		120 (13.3)	25 (11.7)	1 (ref.)	---
4–11 months		274 (30.3)	67 (31.3)	1.2 (0.7–2.1)	0.435
12–59 months		511 (56.3)	122 (57)	1.2 (0.7–1.9)	0.479
Area of residence					
Urban		429 (47.4)	113 (52.8)	1 (ref.)	---
Rural		476(52.6)	101 (47.2)	0.75 (1.2–2.1)	0.071
Sex					
Male		538(59.4)	128 (59.8)	1 (ref.)	---
Female		367(40.2)	86 (40.6)	0.98	0.901
Season***					
Dry season		612 (67.6)	132 (61.7)	1 (ref.)	---
Rainy season		293(32.4)	82 (38.3)	1.4 (1.02–1.9)	0.034
Sites					
Bogodogo		275 (30.4)	120 (56.1)	1 (ref.)	
Bousse		160 (17.7)	3 (1.4)	0.02 (0.007–0.07)	< 0.001**
Kongoussi		224 (24.8)	69 (32.2)	0.57 (0.4–0.8)	0.003
Houndé		246 (27.2)	22 (10.3)	0.12 (0.07–0.21)	< 0.001**
Antibiotics before admission					
No		358 (39.6)	77 (36)	1 (ref.)	---
Yes		547 (60.4)	137 (64)	1.2 (0.9–1.7)	0.221
Antibiotics during hospitalization					
No		17 (1.9)	7 (3.3)	1 (ref.)	0.023*
Yes		888 (98.1)	207 (96.7)	0.4 (0.16–1.15)	0.095
Chronic health condition					
No		894 (98.8)	213 (99.5)	1 (ref.)	---
Yes		11(1.2)	1 (0.5)	0.3 (0.04–2.5)	0.278
Very severe viral ARI					
No		626 (69.2)	152 (71)	1 (ref.)	---
yes		279(30.8)	62 (29)	0.9 (0.6–1.2)	0.501
Delay of consultation*****					
0–2 days		446 (49.3)	102 (47.7)	1 (ref.)	---
3–5 days		345(38.1)	81 (37.9)	1.03 (0.7–1.4)	0.841
6 days and more		114(12.6)	31 (14.5)	1.25 (0.8-2)	0.334
Malnutrition					
No		833 (92)	192 (89.7)	1 (ref.)	---
Yes		72 (8)	22 (10.3)	1.46 (0.9–2.5)	0.152

*p < 0.05 ** *p* < 0.01 VSARI : Viral severe acute respiratory infection OR : odds ratio; ratio ***Rainy season : June to September, dry season : October to may; ****prolonged hospital stay : length of stay > 7 days; ***** Time between the onset of symptoms and the consultation to the hospital

**Table 4:** Univariate analysis of pathogens associated with prolonged hospital stay (> 7 days) of viral severe acute respiratory infections among children under five years of age

Variables	Total Positive (%) *N* = 905	Prolonged hospital stay (%) *N* = 214	Crude OR (95% CI) (Positive vs. Negative)	*p*-value
Respiratory syncytial virus	157 (17.3)	35 (16.4)	0.9 (0.6–1.3)	0.661
Rhinovirus	340(37.6)	88 (41.1)	1.2 (0.9–1.6)	0.220
Parechovirus	10 (1.1)	2 (0.9)	0.8 (0.16–3.8)	0.785
Human parainfluenza virus 1	23 (2.5)	7 (3.3)	1.4 (0.6–3.5)	0.440
Human parainfluenza virus 2	18 (2)	1 (0.5)	0.18 (0.02–1.4)	0.103
Human parainfluenzavirus 3	70 (7.7)	16 (7.5)	0.95 (0.5–1.7)	0.871
Human parainfluenzavirus 4	44 (4.9)	8 (3.7)	0.7 (0.3–1.5)	0.382
Influenza A	126 (13.9)	29 (13.6)	0.95 (0.6–1.5)	0.858
Influenza B	58 (6.4)	20 (9.3)	1.8 (1-3.1)	0.047*
Influenza C	14 (1.5)	1(0.5)	0.2 (0.03–1.8)	0.176
Human metapneumovirus	58 (6.4)	5 (2.3)	0.3 (0.1–0.7)	0.009**
Enterovirus	118 (13)	21 (9.8)	0.7 (0.4–1.1)	0.11
Coronavirus OC43	29 (3.2)	10 (4.7)	1.7 (0.8–3.8)	0.168
Coronavirus NL63	28 (3.1)	4 (1.9)	0.52 (0.2–1.5)	0.244
Coronavirus HKU1	25 (2.8)	5 (2.3)	0.8 (0.3–2.2)	0.664
Coronavirus 229E	8 (0.9)	2 (0.9)	1.1 (0.2–5.4)	0.928
Bocavirus	102 (11.3)	18 (8.4)	0.7 (0.4–1.1)	0.132
Adénovirus	201 (2.2)	44 (20.6)	0.9 (0.6–1.3)	0.507
*Staphylococcus aureus*	160 (17.7)	40 (18.7)	1.1 (0.7–1.6)	0.657
*Klebsiella pneumoniae*	321 (35.5)	90 (42.1)	1.4 (1–2)	0.022*
*Legionella pneumophil /Legionella longbeach*	1 (0.1)	0 (0)	----	----
*Streptococcus pneumoniae*	526(58.1)	107 (50)	0.6 (0.5–0.9)	0.006
*Bordetellaspp*	5 (0.6)	1 (0.5)	0.8 (0.1–7.2)	0.848
*Chlamydia pneumoniae*	2 (0.2)	0 (0)	----	---
*Haemophilus influenzae*	390 (43.1)	71 (33.2)	0.6 (0.4–0.8)	0.001**
*Haemophilus influenzae b*	20 (2.2)	4 (1.9)	0.8 (0.3–2.4)	0.698
*Moraxella catarrhalis*	383 (42.3)	74 (34.6)	0.6 (0.5–0.9)	0.009**
*Mycoplasma pneumoniae*	7 (0.8)	2 (0.9)	1.2 (0.2–6.7)	0.759
*Pneumocystis jirovecii*	22 (2.4)	5 (2.3)	0.9 (0.3–2.6)	0.918
*Salmonella spp*	0 (0)	0 (0)	-----	-----
virus-bacteria codetection				
No	128 (14.1)	38 (17.8)	1 (ref)	
Yes	777 (85.9)	177 (82.2)	0.7 (0.5–1.1)	0.105
Type of bacterial codetection				
No bacteria	128 (14.1)	38(17.8)	1 (ref)	
Mono-infection	209 (23.1)	56 (26.2)	0.9 (0.5–1.4)	0.566
Multiple bacterial codetection	568 (62.8)	120 (56.1)	0.63 (0.4-1)	0.038*
Type of mixed viral infection				
Monoviral	455(50.3)	119 (55.6)	1 (ref)	
Two viruses	310 (34.3)	70 (32.7)	0.8 (0.6–1.1)	0.173
Three and more viruses	139 (15.4)	25 (11.7)	0.6 (0.4–0.9)	0.026*

*p < 0.05 ** *p* < 0.01 ; VSARI : Viral severe acute respiratory infection OR : odds ratio; ****prolonged hospital stay : length of stay > 7 days

## Discussion

Our study focused on two key determinants of the viral SARI burden in children and healthcare systems: the severity and length of stay. We highlighted the important role of some socio-demographic and clinical factors, as well as codetection of other pathogens and occurrence in rainy season in worsening of viral SARI case in children.

Residence in urban areas, age of less than three months, and codetection with *Klebsiella pneumoniae* and *Haemophilus influenzae* type b were associated with increased severity of severe acute respiratory infection. Malnutrition, hospitalization during the rainy season, and infection with human CoronavirusOC43 were significantly associated with prolonged hospitalization among those with viral SARI.

### Increased severity of viral acute respiratory infections

Our study found increased odds of VSVARI among patients from urban areas compared to rural residents. This could be because one of our collection sites was an urban referral teaching hospital that is better equipped and therefore likely to receive patients with severe illness from hospitals that are less equipped with intensive care equipment and have less qualified personnel. Residents living in urban areas may be better able to access hospitals to seek care than those who are very ill and may live a long distance from a rural hospital. However, other studies revealed that the most severe forms of ARI were related to environmental risk factors that are generally more present in urban than rural settings. Cummings et *al.* in a spatiotemporal study in Uganda in 2016 found a higher risk of more severe forms of ARI in urban and peri-urban areas than in rural areas [27]. A study by Akinyemi and Morakinyo in Nigeria in 2018 [28] also implicated the living environment, especially intra-domiciliary pollution, as a risk factor of ARI. Kafando et al. also observed similar findings in the city of Ouagadougou in 2018 [29].

Nevertheless, regional disparities in the distribution of these risk factors must be considered in preventing severe ARIs among children. Several recent studies in Ethiopia [30], Nigeria [14] and Uganda [27] have found that ARI can present substantial regional disparities within a single country because of the differences in the standard of living (poverty) and geo-climatic factors.

Our findings provide further evidence of the association of young age with severe acute respiratory infection. In addition to the immaturity of the child’s immune system and the changing immune processes, the role of infection in the evolution of immune defenses and allergies in children is becoming increasingly evident [31]. The more severe forms of ARIs are typically found among younger children, regardless of specific viral etiology. This trend is also found in most of the studies in the West Africa sub-region [11, 16, 32].

During hospitalization, children who were not treated with antibiotics had 2.7 times greater odds of very severe viral SARI. These results should be interpreted with caution, given the usage of antibiotics in our study population during their hospital stay (98.3% treated with antibiotics). Although it is recognized that the ARIs are predominantly caused by viral infections, antibiotics are widely administered to hospitalized patients in developing countries [33]. It is also worth noting that bacterial codetection in people with viral ARIs is one of the major causes of severity and hospitalization among children [9], hence the frequent use of antibiotics in severe ARI cases.

The presence of a bacterial coinfection in viral ARI is very often a source of greater severity. Viral damage to the epithelial barrier and impaired mucociliary function weakens airway defenses, increasing vulnerability to bacterial coinfection [34]. *Klebsiella pneumoniae* and *Haemophilus influenzae* type b are widely recognized as bacterial etiological agent of many types of infections, including respiratory infections. There are responsible for community-acquired ARI and hospital-acquired forms, which are the most virulent as they are often resistant to the usual antibiotics [35]. *Haemophilus influenzae type* b is included in the Burkina Faso Expanded Program on Immunization. The detection of this pathogen and its association with SARI raise questions regarding vaccine coverage, warranting further investigations [36]. Furthermore, in recent years, new subtypes of *Klebsiella pneumoniae* responsible for very severe respiratory infections have been reported [35], including the hypermucoviscous form described as hypervirulent in many Asian countries and increasingly in Europe, with limited data available in Africa [37–39]. Our results also demonstrate, multiple bacterial co-infections increased the risk of VSVARI occurrence. In the majority of similar studies conducted in the sub-region, bacterial co-infection is identified as a contributing factor to the severity of viral Acute Respiratory Infections (ARIs) [9, 17].

However, it is crucial to keep in mind that the sampling and diagnostic methods used in our study, such as oro/nasopharyngeal swabbing and rRT-PCR, may detect carrier organisms that are not necessarily the etiology of the SARI [40].

### Factors associated with prolonged hospitalization of viral SARI

In multivariable analysis, we found that malnutrition, rainy season, infection with Coronavirus OC43, influenza B, hMPV AB, and *Klebsiella pneumoniae* were associated with prolonged hospitalization (> 7 days) in children under five years of age. Numerous studies have reported the effect of malnutrition (severe and moderate forms) on susceptibility to several types of infection. Bryce et *al.*, found malnutrition to be the main mortality factor in 52.3% of children with pneumonia [41]. The relationship between malnutrition and acute infection disease is bidirectional: malnutrition increases risk of infection, and the infection worsens the malnutrition state [42]. Ngari et *al.* [43], and Lazzeri et *al.* [44], in recent studies in Kenya and Malawi, respectively, found a strong association between malnutrition and mortality from ARI. Cox et *al.*, in Malawi, found malnutrition to be a predictor of the development of respiratory infection [13]. However, we did not find a study in the literature that specifically explored the relationship between respiratory viral infection, length of hospitalization, and malnutrition.

Children admitted to the hospital during the rainy season for viral SARI were 1.6 times more likely to have a prolonged hospitalization than those hospitalized during the dry season. Annual peaks in the incidence of the viruses responsible for ARI in tropical regions are primarily observed in the dry season [45, 46], even though some studies report less evidence of seasonality for several respiratory pathogens in tropical areas [47]. Malaria is highly prevalent in children under five in the rainy season in Burkina Faso, and the comorbidity with ARI could contribute to a higher risk of a lengthy hospital stay [22, 23]. In addition, the rainy season in rural areas corresponds to a “lean season”. Food stocks in rural areas are generally at their minimum, exposing the younger population to malnutrition. Nevertheless, the absence of data on malaria comorbidity does not allow us to refine our analysis.

Children infected with influenza B virus, Coronavirus OC43, and *Klebsiella pneumoniae* had an increased risk of prolonged hospitalization in our population, whereas infection with human metapneumovirus decreased this risk. Influenza B virus is poorly studied compared with influenza A. Therefore, there are still many unclear areas and gaps in knowledge about its epidemiology and pathogenicity [48], even though clinically, influenza B virus infection shows few differences with influenza A and other respiratory viruses. Nevertheless, it has been reported that influenza B virus infection has been associated with severe ARI in children, leading to admission to intensive care units with a risk of prolonged hospitalization [49]. In our study, the odds of prolonged hospitalization were twice as high in the patients with influenza B virus infection in multilevel multivariable analysis, reflecting a relatively strong association. However, little is known about the immunological mechanisms and virological characteristics that may explain the higher pathogenicity of the influenza B virus compared to influenza A, and very few studies have been conducted [17].

As for Human coronavirus OC43, its association with prolonged hospitalization found in our study is not common in the literature. Indeed, among the six coronaviruses type responsible for respiratory infection in humans before the advent of SARS-CoV-2 in 2020 in Burkina Faso [40], only MERS-Cov and SARS-Cov were implicated in the most severe forms of ARI in many countries. The other types of coronaviruses are most often responsible for less severe cases or even mild rhinitis. Nevertheless, the presence of Human coronavirus OC43 as an etiological agent of SARI has been described in numerous studies in sub-Saharan Africa without association with possible severity or prolonged hospitalization of cases being investigated [50–52]. The larger size of our sample, the possible presence of undetected comorbidities, and our analysis methods (multilevel logistic regression) may explain our findings.

*Klebsiella pneumoniae* was the only pathogen associated with the severity of ARI and prolonged hospitalization. Nevertheless, as described above, there may be interdependence between severe ARI and the risk of prolonged hospitalization. The possible existence of nosocomial infections, the resistance to classical antibiotics, and the virulence of certain subtypes of *Klebsiella pneumoniae* may explain our results [35, 53].

### Strengths and limitations

The main strength of our study was the use of multi-pathogen screening allowing the detection of a wide range of viral respiratory pathogens with the advantage of the high sensitivity of PCR techniques combined with a quick turn-around time to results. To our knowledge, this is the first research of this kind in Burkina Faso. The combination of the detection of bacterial and fungal pathogens allowed the identification of possible coinfections. Furthermore, use of the standardized WHO SARI case definition allows us to compare across our study population and with other surveillance systems worldwide. Similarly, our study sites, located in both urban and rural areas, ensured heterogeneity in the study population.

Nevertheless, our study had limitations related to the nature of cross-sectional studies. Pathogens found in nasal and/or oropharyngeal samples may not match those that are inside the lung, therefore, some detected germs may not necessarily be related to the patient’s symptomatology. Moreover, the absence of certain variables, particularly those related to malaria and other comorbidities, the impossibility to assess the temporality of some explanatory variables compared with the dependent variables (such as the co-infection) and the non-systematic measure of the oxygen saturation rate did not allow us to refine our severity analysis.

## Conclusion

This study was motivated by the significant impact of viral SARI on children’s morbidity and mortality and the relatively limited data on the subject in the African sub-region in general and in Burkina Faso. Our report is the first that identifies some specific and atypical pathogens in SARI in Burkina Faso. The results suggest the need for practitioners in Burkina Faso and other countries sharing similar features to pay more attention to early diagnosis and management of comorbidities such as malnutrition in children hospitalized with SARI particularly during malaria peak seasons to prevent complications. Additionally, implementation of public health policies for early etiological diagnosis and management of certain forms of viral ARI including vaccination are important to control ARI morbidity and mortality in children. The results also call for a further investigation of comorbidities such as malaria, and a better understanding of bacterial coinfection and their role in the morbidity and mortality of viral SARI in children.

### Electronic supplementary material

Below is the link to the electronic supplementary material.

Supplementary Material 1

## Data Availability

The datasets generated and analysed during the current study are available from the corresponding author on reasonable request.
