# Supplementary material for: Predictors of severity and prolonged hospital stay of viral acute respiratory infections (ARI) among children under five years in Burkina Faso, 2016–2019
Source: BMC Infect Dis. 2024 Mar 20;24:331. doi: 10.1186/s12879-024-09219-x (PMC10953152; doi:10.1186/s12879-024-09219-x)
Supplement: Supplementary file 1 — Supplementary Material 1 [file 12879_2024_9219_MOESM1_ESM.docx]

**ANNEXES**

**ANNEX 1:** Output of the null two-effect model with random intercepts and fixed effects of the hospital length of stay analysis

***Interpretation*** *The test statistic is 139.45 with a corresponding p-value of less than 0.05 (0.012) and so there is strong evidence that the between hospital variance is non-zero*

**Figure S1:** Output of the null two-effect model with random intercepts and fixed effects of the hospital length of stay analysis

**ANNEX 2:** Specification tests for Models

***Interpretation:*** *The link test reveals no problems with our specification (hat significative and hat square not significative using p<0.05 as a level*

**Figure S2:** STATA Output of the linktest for the model specification on “ Very severe viral acute respiratory infection compared to other SARI cases”

***Interpretation:*** *The overall rate of correct classification is estimated to be 72.4%, with 93.71% of the normal weight group correctly classified (specificity) and only 21.7% of the low weight group correctly*

*classified (sensitivity).*

**Figure S3:** STATA Output of the post-estimation classification table for the model “Very severe viral acute respiratory infection compared to other SARI cases” validation

**Figure S 4:** Graphic of the area under the Received operating procedure (ROC) curve of the residual of the mixed multilevel model “length of hospital stay”

***Interpretation:*** *the postestimation test of the mixed multilevel model “length of hospital stay” show an area under the ROC curve of the residuals = 81.27% which is a high goodness of fit of the model*
